# Hybrid seton for the treatment of high anal fistulas: results of 128 consecutive patients

**DOI:** 10.1007/s10151-013-1021-z

**Published:** 2013-04-30

**Authors:** B. Ege, S. Leventoğlu, B. B. Menteş, U. Yılmaz, A. Y. Öner

**Affiliations:** 1 Department of Surgery, Private Koru Hospital, Ankara, Turkey; 2 Department of Surgery, Gazi University Medical School, 30. Sokak 40/1 Emek, 06510 Ankara, Turkey; 3 Department of Radiology, Gazi University Medical School, Ankara, Turkey

**Keywords:** Fistula-in-ano, High anal fistula, Seton, Hybrid seton

## Abstract

**Background:**

The aim of this study was to evaluate our experience in managing high anal fistulas with a simple modification of the cutting seton.

**Methods:**

We performed a retrospective review of standardized patient charts and of prospectively collected scores and questionnaires. Surgical outcomes of 128 consecutive, well-documented patients with high anal fistulas, including anterior transsphincteric fistulas in females, treated using a hybrid seton, were analyzed.

**Results:**

No significant complications occurred. The mean postoperative pain scores on a visual analog scale were 3.23 and 0.61, on days 1 and 7, respectively. Complete healing was achieved in 67 cases (52.3 %) at 1 month and in all cases (100 %) at 3 months. Recurrent fistula was noted in 2 patients (1.5 %) at 6 and 12 months. The mean postoperative incontinence scores at 3 and 12 months did not differ significantly from the preoperative score (*p* = 0.061, Wilcoxon’s test). The depression, life style, and embarrassment item scores of the fecal incontinence quality of life index improved significantly after surgical treatment.

**Conclusions:**

The results of this series suggest that the hybrid seton might be a valid alternative for the treatment of high anal fistulas, eliminating the need for postoperative adjustments. The slow and stable cutting of the sphincter seems to have a positive effect on the maintenance of continence. The successful outcome is associated with significant improvement in quality of life.

## Introduction

As simple, low anal fistulas can be treated safely only by fistulotomy, the management of complex fistulas needs to balance the outcomes of cure and continence. There is a risk of sphincter muscle damage during fistulotomy, and this might lead to an unacceptable risk of anal incontinence (AI) of varying degrees [1–3]. The degree of incontinence depends on the amount of damaged muscle, preexisting sphincter damage, and scarring of the anal canal. Several alternative treatment strategies have been practiced in order to preserve the sphincter mechanism, including draining setons, cutting setons [3–8], rectal mucosal or full-thickness advancement flaps [9–11], rerouting [12], two-stage seton fistulotomy [13], fistulectomy, anal fistula plug [14–16], ligation of the intersphincteric fistula tract (LIFT) [17, 18], fistulotomy with reconstruction of the sphincter mechanism [19], or fibrin glue [20].

The oldest and theoretically the simplest technique is to use a seton, the well-known variations in modern surgical practice being cutting setons, drainage setons, and two-stage seton fistulotomy [4]. The long-term loose draining seton, while not placing the sphincter at risk, is simply palliative with only marginal rates of complete healing [1, 7]. The tight or cutting seton, on the other hand, has resulted in unacceptable rates of both severity and frequency of AI [21–26]. Therefore, there is an ongoing need to reduce the complications associated with seton use and improve certain weak points of this common technique, such as the above-mentioned high rates of continence disturbances, prolonged discharge and interference with the patient’s quality of life, numerous visits to check and adjust the seton, and staged surgical procedures [4, 13, 21, 27]. In this study, we evaluated our experience in managing high anal fistulas with a simple modification of the cutting seton.

## Materials and methods

### Patient selection

Demographic characteristics, operative findings, and surgical outcomes of the patients were recorded on a standardized form for prospective analysis. Patients with fistulas secondary to inflammatory bowel disease, past or present malignancies, and trauma were excluded from the study, as were patients with a history of vaginal deliveries involving tears, episiotomies or forceps. The other exclusion criteria were the following: horseshoe fistulas, multiple fistulas, or the need for additional concomitant anorectal surgery. Especially in cases of recurrent fistulas, colonoscopy was done in the early preoperative period to rule out Crohn’s disease, and pelvic phase array magnetic resonance imaging (MRI) and/or anal ultrasound was performed as well. The treatment method was decided on during the operation based on the relationship of the fistulous tract with the sphincter muscles. The indication for the use of the elastic cutting seton in this study was a single fistula involving more than half of the sphincter muscles or an anterior transsphincteric fistula in a female patient.

### Surgery

**Fig. 1:**
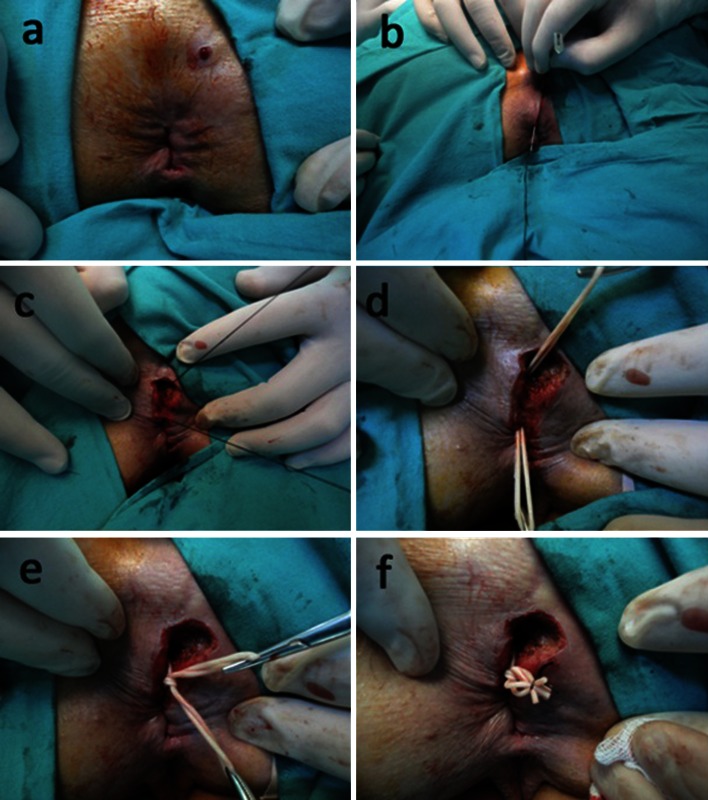
Preoperative view (**a**); mapping of the fistulous tract (**b**); insertion of the silk suture (**c**); positioning of the hybrid seton with the help of the silk suture and incision of the skin and anoderm overlying the fistulous tract (**d**). The hybrid seton was tied over itself for stable and slow cutting (**e**), and the excess ends were trimmed (**f**)

All operations were performed by at least one of the three colorectal surgeons (BBM, SL, BE) with the patient in the prone jackknife position. All operations (except in 3 patients with previous spinal surgery, previous headache after spinal anesthesia, or unsuccessful prior treatment of their fistula) were performed under spinal anesthesia. Application of the hybrid seton was mentioned in our previous pilot study [6], and the surgical steps are shown in Fig. 1a–f. In brief, the fistulous tract was gently probed with a small, blunt-tipped, flexible metal probe. The hybrid seton was created by cutting a thin (2–3 mm) circular strip from a surgical glove (No. 8 latex surgical glove, Beybi^®^, Beybi Plastic Factory, İstanbul, Turkey), including its thicker sleeve. The portion of the tract outside the sphincters was laid open and curetted, as were any lateral secondary tracts. The hybrid seton was tied to the tip of the metal probe and inserted through the remaining tract in a double-strand fashion, or it was drawn into position using a 2/0 silk suture. The skin and anoderm overlying the fistulous tract were incised. This double-strand elastic seton was then tied over itself on the sphincter without excessive tension. Postoperatively, gentle cleaning of the anal region with warm water after each bowel movement was advised. The patients were informed in detail about the presence of the rubber prosthesis, and they were warned about the possible serous discharge that would continue until the seton dropped, and the wound healed.

### Evaluation of the end points

Postoperatively, the patients were reexamined at 7 and 30 days, 3 and 12 months, and then on a yearly basis. However, they were encouraged to contact us whenever they suspected a problem like recurrence and whenever they recognized that the seton dropped. Postoperative pain was evaluated with a visual analog score (VAS) on the first and seventh postoperative days. AI scores were assessed with the Cleveland Clinic Incontinence Score (CCIS) system and quality of life with the Fecal Incontinence Quality of Life Index (FIQLI) preoperatively at admission and postoperatively at 3 and 12 months [28, 29]. The days required for the hybrid seton to cut through and drop were recorded, as well as complications and complete healing rates at 1 and 3 months, respectively. The patients with a CCIS of >1 after the operation were evaluated by anal manometry and endoanal ultrasonography. Recurrence was defined as reappearance of the fistula or development of an additional fistula at or close to the original tract.

Statistical analyses were performed by SPSS 15.00 (SPSS Inc., Chicago, IL, USA). The results were expressed as mean ± SD (standard deviation). The Wilcoxon signed-rank test was used to compare the CCIS, FIQLI scores. The *p* value <0.05 was accepted as statistically significant.

## Results

Between April 2005 and September 2010, 147 consecutive patients fulfilling the above-mentioned selection criteria were treated with the hybrid seton for high anal fistulas. With the loss of 19 patients during follow-up, 128 well-documented patients were analyzed. Ninety-five men (74.2 %) and 33 women (25.8 %), ranging in age from 21 to 74 years (median 45 years) were followed for at least 12 months (range 12–65; median 31 months). Thirty-seven patients (28.9 %) had anterior transsphincteric fistulas (high and/or transsphincteric female). The common symptoms of purulent discharge, pain, and/or pruritus had existed for a median of 15 months (range 1–120 months). Twenty-five of the patients (19.5 %) had undergone fistula surgery 1–4 times.

**Fig. 2:**
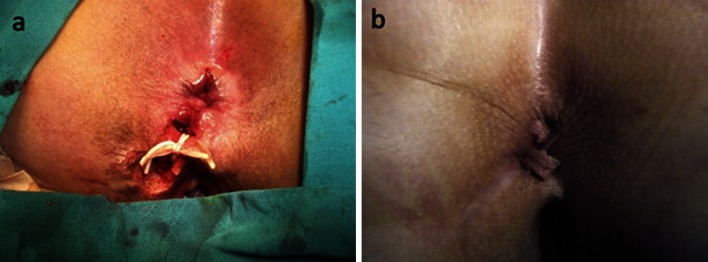
Application of the hybrid seton in a female patient with anterior transsphincteric fistula (**a**). Healing at 3 months with perfect preservation of the contour of the anus (**b**)

**Fig. 3:**
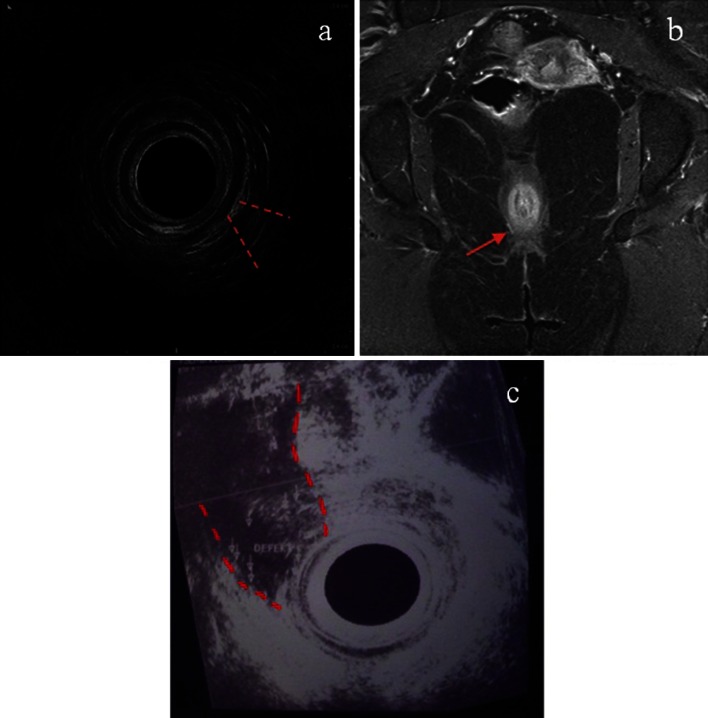
The postoperative anal ultrasound and pelvic magnetic resonance imaging views of patients treated with the hybrid seton (**a**, **b**). Note the limited scarring and dehiscence of the sphincter mechanism when the hybrid seton is used, in contrast with the wide defect caused by staged fistulotomy (**c**), (the sphincter defects are *highlighted* with *red lines* or *arrow*)

All of the patients were discharged on the first or second postoperative day. None required readmission or needed narcotic analgesics after discharge. No infective complications or significant bleeding were noted. There were only 6 cases (4.7 %) of transient urinary retention which required single catheterization. The mean VAS pain scores of the patients on the first and seventh postoperative days were 3.23 and 0.61, respectively. The mean time for the hybrid seton to cut through the sphincter and drop was 18.81 ± 2.80 days (range 11–30 days). In 5 patients, the hybrid seton got loose and trapped beneath the anoderm, and it was easily removed on the 30th day. Complete healing was achieved in 67 cases (52.3 %) at 1 month and in all cases (100 %) at 3 months (Fig. 2a, b). Recurrent fistula was noted in 2 patients (1.5 %) at 6 and 12 months (Fig. 3).

The CCIS and FIQLI results are listed in Table 1. As shown, the mean postoperative incontinence scores at 3 and 12 months did not differ significantly from the preoperative score (*p* = 0.061, Wilcoxon’s test). Worsening of the incontinence score was noted in 7 (5.46 %) patients. In 5 of them, the postoperative CCIS was only 1, and further work-up or treatment was not indicated. In 2 patients (one of them a recurrent case), the preoperative CCIS of 0 changed at 3 months postoperatively to 3 and 5 (excluded in 12-month’s analysis). These 2 patients were evaluated by endoanal ultrasonography and anal manometry and then treated by posterior tibial nerve stimulation. They have managed well within the time limits, and further treatment has not yet been planned.

**Table 1:** Pre- and postoperative CCIS and FIQLI item scores of the patients

Item	Preoperative (mean ± SD)	Postoperative 3rd month (mean ± SD)	Postoperative 12th month (mean ± SD)	*p*
CCIS	0.40 ± 0.51	0.42 ± 0.80	0.42 ± 0.57	0.061
FIQLI- lifestyle	3.98 ± 0.03	3.997 ± 0.04	4.00	0.004*
				0.001**
FIQLI-coping/behavior	3.99 ± 0.03	4.00	4.00	0.102
FIQLI-depression/self-perception	3.90 ± 0.13	3.99 ± 0.05	4.00	0.0001*
				0.0001**
FIQLI-embarrassment	3.97 ± 0.03	3.99 ± 0.12	3.99 ± 0.12	0.002*
				0.0001**

Comparisons were performed by the Wilcoxon signed-rank test

* Preoperative versus postoperative 3rd month

** Preoperative versus postoperative 12th month

As also seen in the table, the FIQLI scores also improved after treatment. The depression, life style, and embarrassment scores improved significantly (Table 1). The elimination of the disease process with continuous leakage and pain possibly contributed to the improvement in these item scores. The improvement was evident at both 3 and 12 months, postoperatively. Therefore, it seems that the hybrid seton did not negatively influence quality of life (QoL).

## Discussion

This study has shown that hybrid seton is an effective tool for the treatment of high anal fistulas. The median follow-up period is longer than 2 years, and the success rate appears to be high. Following on our pilot study, this is a study performed in a relatively large patient group with longer follow-up [6]. There were only 2 cases of recurrence within the time limits. Moreover, complete healing was achieved in all cases at 3-months. The study naturally suffers from being a single-armed series including selected cases. The difficult target is the complex fistula, that is, those fistulas with any of these characteristics: primary track crossing 30–50 % of the external sphincter (high-transsphincteric, suprasphincteric, and extrasphincteric), anterior track in a female, multiple tracks, recurrent fistula, and fistula in a patient with preexisting incontinence, previous local irradiation, chronic diarrhea, or Crohn’s disease. Based on the promising results obtained with our patients fulfilling some of these criteria, further investigations may include patients with inflammatory bowel disease or other risk factors for anal incontinence. Furthermore, patients with simple anal fistulas and risk factors for anal incontinence might also benefit from this technique.

The use of the hybrid seton was not associated with any significant complications or significant pain. The postoperative VAS scores were low, and none of the patients needed narcotic analgesics after discharge. It is probable that the soft and elastic features of the hybrid seton prevented possible postoperative pain or discomfort encountered when rigid materials are used. In addition, the simple, original technique described in this study precludes the need for any additional maneuvers or adjustments performed by the patient or the surgeon, which inevitably bring about further visits and additional pain. The slow and stable cutting action on the sphincters eventually results in a primary fistulotomy, rather than a staged procedure.

There exist numerous surgical techniques for the treatment of high/complex anal fistulas, but none has proven to be ideal. For example, although fibrin glue has been used in the treatment of high fistula, a notable series is unlikely to appear, based on the low-healing rates reported with this method [30–32]. The LIFT technique, which aims at total anal sphincter preservation, appears to be both safe and easy to perform. The healing rate ranges from 57 to 94 %, and it seems to effectively preserve continence [17, 18]. However, the efficacy and durability of LIFT should be assessed in long-term follow-up. The reported recurrence rate varies as the technique is operator dependent [17]. Moreover, the efficacy of LIFT has not been proven specifically in Crohn’s disease in which drainage is of primary importance.

Therefore, seton techniques still occupy an important position in the treatment of high anal fistulas. Of the various seton techniques used in modern surgical practice, drainage setons are far from providing a radical cure, and they are mainly useful in patients with chronic sepsis secondary to perianal Crohn’s disease or acquired immune deficiency syndrome-related anorectal sepsis [1, 4, 7]. A cutting seton works by slowly transecting the enclosed sphincter muscle as a result of pressure necrosis with the hope of minimal separation of the cut ends. After the first report of Hanley, several setons and tightening techniques, including silk, rubber braided silk, rubber band, chemically treated linen, silastic, Penrose drain, and elastic, nylon have been used [26, 33]. The tightening was performed every second day, every week, every month, etc. [26]. Hanley first reported the use of a rubber band seton in the surgical management of anterior abscess-anal fistula and anterolateral fistula in women, with good functional results [33]. In his study, the elastic rubber seton was intermittently tightened with silk ligatures. In 1984, Culp described the use of a thin Penrose drain as a primary operative seton [34]. All 20 patients had good results with little or no postoperative discomfort and with only 3 cases of minor incontinence. Once again, tension was maintained by a tie of heavy silk suture, and postoperatively, the patient was instructed to grasp the ends of the elastic seton and move it several times daily. Dziki and Bartos reported good results with a similar rubber band seton which was tightened around the external sphincter by a thread tied around its ends [35]. More recently, Hammond and coworkers reported the use of a 1-mm silastic seton which was ‘snugly’ tied around the sphincter [36]. In their series of 29 patients, healing was achieved in 100 % with minor incontinence in 25 %. Our previous series of horseshoe fistulas treated with the hybrid seton has also confirmed the advantages of this application [37]. In our series, the slow, stable, and continuous ‘cut through’ provided by the hybrid seton led to a high healing rate with minimal postoperative morbidity. No objective assessment has been made for the degree of tension imposed by the hybrid seton.

Another major issue regarding the hybrid seton was that worsening of continence was noted in only 7 (5.46 %) patients. The contour of the anus was largely preserved, and baseline continence was not significantly disturbed. The possible advantages of the technique include both the ability to drain the region with the prevention of recurrent abscess and the promotion of fibrosis around the seton during slow division of the sphincter. As Hammond suggested, the formation of fibrosis prevents retraction of the sphincter behind the seton’s advance. The favorable results obtained with the hybrid seton might stem from the fact that an intermittently tightened static seton causes intermittent sharp cuts, while the cutting provided by the hybrid seton is slow and stable. This feature may be particularly valuable considering that a recent review on cutting setons revealed that incontinence rates ranged from 31 to 53 %, and the overall anal incontinence rate was 12 % when any kind of seton was used [26]. Alternatively, two-stage seton fistulotomy is a widely practiced method which utilizes the seton to mark the tract and stimulate fibrosis, followed by delayed incision of the sphincters as a second procedure. This method is, by definition, a staged procedure, and it is not free from significant disturbances in anal continence [13, 27, 38].

Our study showed that the patients’ social parameters and some psychological parameters were disturbed preoperatively because of the symptoms of fistula and/or the knowledge of having a chronic and poorly treated disease, as has been previously suggested [39]. For this evaluation, we preferred to use the FIQLI to better address the impact of the most feared complications of fistula surgery. The study of Garcia et al. [40] showed that soiling, gas incontinence, and recurrences disturbed patients’ QoL following surgery. Parameters related to lifestyle limitations, depression, and embarrassment all developed significantly after successful surgery with our technique. The nonsignificant improvement in the symptom scores related to preoperative discharge, pain and itching associated with the fistula might suggest that preoperative QoL was more profoundly influenced by psychological and social problems, compared with the physical sequelae. The successful clinical outcome in our series, with a high healing rate and low recurrence and complication rates correlated with the improvement in QoL. It is possible that similar improvements could be found using other QoL assessment tools.

## Conclusions

The long-term results of this series have suggested that the hybrid seton might be a valid alternative for the treatment of high anal fistulas. The possible positive contribution of the slow and stable cutting of the sphincter on the maintenance of continence is also demonstrated. An important practical disadvantage of conventional seton treatment, namely the need for postoperative adjustments, is eliminated. In spite of the perfect preservation of continence in the short term in our series, possibly due to the slower, stable, and gradual severance of the sphincters by the hybrid seton, it would be reasonable for our colleagues to adopt this technique initially in patients without any identifiable past or future risk of incontinence until they personally observe the different mechanism of the hybrid seton and larger series with longer follow-up accumulate. It is probable that seton treatment for anal fistula will evolve with further comparative studies and technical innovations.
